# Behavioral and Cardiorespiratory Responses to Bilateral Microinjections of Oxytocin into the Central Nucleus of Amygdala of Wistar Rats, an Experimental Model of Compulsion

**DOI:** 10.1371/journal.pone.0099284

**Published:** 2014-07-18

**Authors:** Érica Maria Granjeiro, Simone Saldanha Marroni, Daniel Penteado Martins Dias, Leni Gomes Heck Bonagamba, Kauê Machado Costa, Jéssica Cristina dos Santos, José Antônio Cortes Oliveira, Benedito H. Machado, Norberto Garcia-Cairasco

**Affiliations:** 1 Department of Physiology, Ribeirão Preto School of Medicine, University of São Paulo, Ribeirão Preto, SP, Brazil; 2 Department of Neuroscience and Behavioral Sciences, Ribeirão Preto School of Medicine, University of São Paulo, Ribeirão Preto, SP, Brazil; The University of Manchester, United Kingdom

## Abstract

**Introduction:**

The central nucleus of amygdala plays an important role mediating fear and anxiety responses. It is known that oxytocin microinjections into the central nucleus of amygdala induce hypergrooming, an experimental model of compulsive behavior. We evaluated the behavioral and cardiorespiratory responses of conscious rats microinjected with oxytocin into the central nucleus of amygdala.

**Methods:**

Male Wistar rats were implanted with guide cannulae into the central nucleus of amygdala and microinjected with oxytocin (0.5 µg, 1 µg) or saline. After 24 h, rats had a catheter implanted into the femoral artery for pulsatile arterial pressure measurement. The pulsatile arterial pressure was recorded at baseline conditions and data used for cardiovascular variability and baroreflex sensitivity analysis. Respiratory and behavioral parameters were assessed during this data collection session.

**Results:**

Microinjections of oxytocin (0.5 µg) into the central nucleus of amygdala produced hypergrooming behavior but did not change cardiorespiratory parameters. However, hypergrooming evoked by microinjections of oxytocin (1 µg) into the central nucleus of amygdala was accompanied by increase in arterial pressure, heart rate and ventilation and augmented the power of low and high (respiratory-related) frequency bands of the systolic arterial pressure spectrum. No changes were observed in power of the low and high frequency bands of the pulse interval spectrum. Baroreflex sensitivity was found lower after oxytocin microinjections, demonstrating that the oxytocin-induced pressor response may involve an inhibition of baroreflex pathways and a consequent facilitation of sympathetic outflow to the cardiovascular system.

**Conclusions:**

The microinjection of oxytocin (1 µg) into the central nucleus of amygdala not only induces hypergrooming but also changes cardiorespiratory parameters. Moreover, specific oxytocin receptor antagonism attenuated hypergrooming but did not affect pressor, tachycardic and ventilatory responses to oxytocin, suggesting the involvement of distinct neural pathways.

## Introduction

Grooming is a spontaneous behavior that occurs widely in many species [1], [2] and is associated, among others, with the development of mammary glands, fur cleaning, social communication, temperature regulation and dearousal [1], [2]. Grooming behavior is usually found in three contexts: as a direct reaction to peripheral stimulation and/or contamination (skin); as a displacement behavior, occurring in situations in which the animal experiences behavioral conflict or indecision; and as a reaction to recent arousal, waking up or similar stressful situations [2].

Several peptides, of both hypophyseal [3] and extrahypophyseal [4] origin, can induce exacerbated grooming behavior (hypergrooming). A recent study from our laboratory [5] demonstrated that bilateral microinjections of oxytocin (OT) into the central nucleus of amygdala (CeA) of Wistar rats induced hypergrooming, which we proposed as an experimental model of compulsive behavior [5]. This behavioral response is dependent on the activation of oxytocinergic afferents from the hypothalamic grooming area, which consists of parts of the paraventricular hypothalamic nucleus (PVN) and dorsal hypothalamic area. This hypothesis is corroborated by the co-localization of OT and its receptors in retrogradely labeled cells in these regions [5]. Considering that OT has an excitatory role on PVN oxytocinergic neurons [6], [7], it was suggested that stimulation of PVN OT receptors might induce a release of OT in the CeA and subsequently facilitate the induction of grooming behavior [5].

A major obstacle for understanding grooming behavior is that the neuronal circuitry that generates these behavioral patterns does not seem to be organized in discrete centers, but is rather arranged in a complex integrative network [8], [9]. It is plausible to speculate that the activation of this system may not only induce behavioral responses but also many other physiological alterations. Previous studies demonstrated that CeA also modulates autonomic and neuroendocrine functions [10]. Electrical or chemical stimulation of CeA in rats and cats produce an increase in heart rate (HR), arterial blood pressure (BP) and muscle blood flow [11]–[13]. In addition, there is evidence that baseline respiratory rhythm is also influenced by the CeA [14].

While previous studies have investigated the cardiovascular and respiratory responses induced by CeA stimulation [10], [11], [13], [15], the role of this area in the integration of behavioral and cardiorespiratory responses remains uncertain. This is an important issue, as human patients with behavioral disorders have a significantly higher incidence of cardiovascular diseases [16]–[18]. However, no experimental study has systematically assessed the possible cardiovascular and respiratory responses associated with compulsive behavior.

Considering that CeA is a limbic structure which has a direct influence on behavioral, autonomic, neuroendocrine and respiratory functions and the important role of OT in cardiorespiratory control [19], [20], we hypothesized that hypergrooming induced by bilateral microinjections of OT into the CeA of rats is accompanied by autonomic and respiratory responses. For this purpose, we simultaneously evaluated the behavioral and cardiorespiratory responses in this experimental model of compulsive behavior.

## Methods

### Ethics Statement

The experimental protocols carried out in the current study were approved by the Committee on the Ethics of Animal Experimentation from the Ribeirão Preto School of Medicine (Protocol n° 080/2009). In addition, all efforts were made in order to eliminate or minimize suffering of animals involved.

### Animals

Male Wistar rats (n = 36), 270-310 g, obtained from the Animal Facility of the Campus of Ribeirão Preto, University of São Paulo, Brazil, were housed under controlled conditions of light (lights on, 06:00 hours; lights off, 18:00 hours) and temperature (23±2°C), with access to food and water *ad libitum*. Rats were assigned to the following groups: (I) saline (SAL/inside CeA); (II) OT 0.5 µg/inside CeA; (III) OT 1 µg/inside CeA; (IV) OT 1 µg/outside CeA; (V) SAL+OT 1 ug/inside CeA and (VI) OT antagonist vasotocin (OTA 1 µg+OT 1 ug/inside CeA). It is noteworthy to mention that rats were allowed to rest quietly into the recording room before data collection, and behavioral and cardiovascular parameters were measured at the same time. Additional details in Text S1.

### Implantation of guide cannulae and catheter

Four days prior to the experiments rats were anesthetized with tribromoethanol (2.5%, 1 mL/100 g/i.p.; Sigma–Aldrich, MO, USA) and stainless steel guide cannulae were stereotaxically implanted bilaterally into the CeA.

One day prior to the experiments rats were anesthetized with tribromoethanol and a catheter was implanted into the abdominal aorta through the femoral artery (PE-10 connected to PE-50 tubing, Clay Adams, Parsippany, NJ, USA) for BP measurement. Further details in Text S1.

### Microinjections into the CeA of rats

All microinjections into the CNS were performed with the rat inside a whole-body plethysmographic chamber without any restraint [21], [22]. The SAL or OT (both 200 nL) was delivered into the CeA with a 5 µL syringe (Hamilton, Reno, NV, USA). Further details in Text S1.

### Ventilatory analysis

Measurements of ventilatory parameters [respiratory frequency (f_R_), tidal volume (V_T_) and minute ventilation (V_E_)] were performed using the whole-body plethysmographic method described by Bartlett and Tenney [23] and adapted to our experimental conditions in accordance with detailed information already published [21], [22], [24]. To reach this goal, respiratory oscillations during grooming behavior were not considered for data analysis.

Each animal was placed inside an opened plethysmographic chamber and allowed to adapt for at least 30 min. During this period, the femoral artery catheter was exteriorized throughout a little hole in the top of the chamber, which was sealed with silicone grease during recording, allowing simultaneous recording of cardiovascular and respiratory parameters. Next, the plethysmographic chamber was closed for 2 min and baseline pulsatile arterial pressure, f_R_, V_T_ and V_E_ were acquired (control). Following chamber opening, bilateral microinjections of OT or SAL into the CeA were performed and cardiovascular parameters and grooming behaviors were then recorded during 60 min. The plethysmographic chamber was closed again at 5, 20, 40 and 60 min after the microinjections and ventilatory parameters were recorded. Further details in Text S1.

### Behavioral and Statistical Analysis

Grooming behaviors were quantified using an observational method described by Gispen et al. [25]. Videotaping began when the animal received CeA bilateral microinjections of OT (0.5 µg or 1 µg) or SAL and the rat was then observed during 60 min (details in the Supporting Information). Behavioral data are shown as means and were analyzed using a Poisson model [5], [26]. Considering the grooming count as a dependent variable, a longitudinal Poisson model regression with random effects [27] was used to estimate the means at each time and compare them. In all statistical analyses the level of significance was set at p<0.05.

Neuroethological statistical analysis of the behavioral flowchart (Figure S4) was perfomed and arrows represent statistical values (X^2^> 3.84; p<0.05) highlighting the strength of association between pairs of behaviors (dyads).

The cardiorespiratory data were expressed as the mean ± standard error of mean (SEM). Mean arterial blood pressure (MABP), HR, f_R_, V_T_ and V_E_ changes were analyzed using two-way ANOVA with treatment as independent factor and time as repeated measurement. Data of spontaneous baroreflex sensitivity (BRS) and cardiovascular variability analysis were analyzed by one-way ANOVA with treatment as independent factor. When differences were found, a post-hoc Bonferroni's test was performed.

## Results

### Histological analysis

Figure S1 (Text S1) shows the sites of bilateral microinjections of OT (0.5 µg, OT 1 µg) or SAL.

### Behavioral analysis

**Figure 1:**
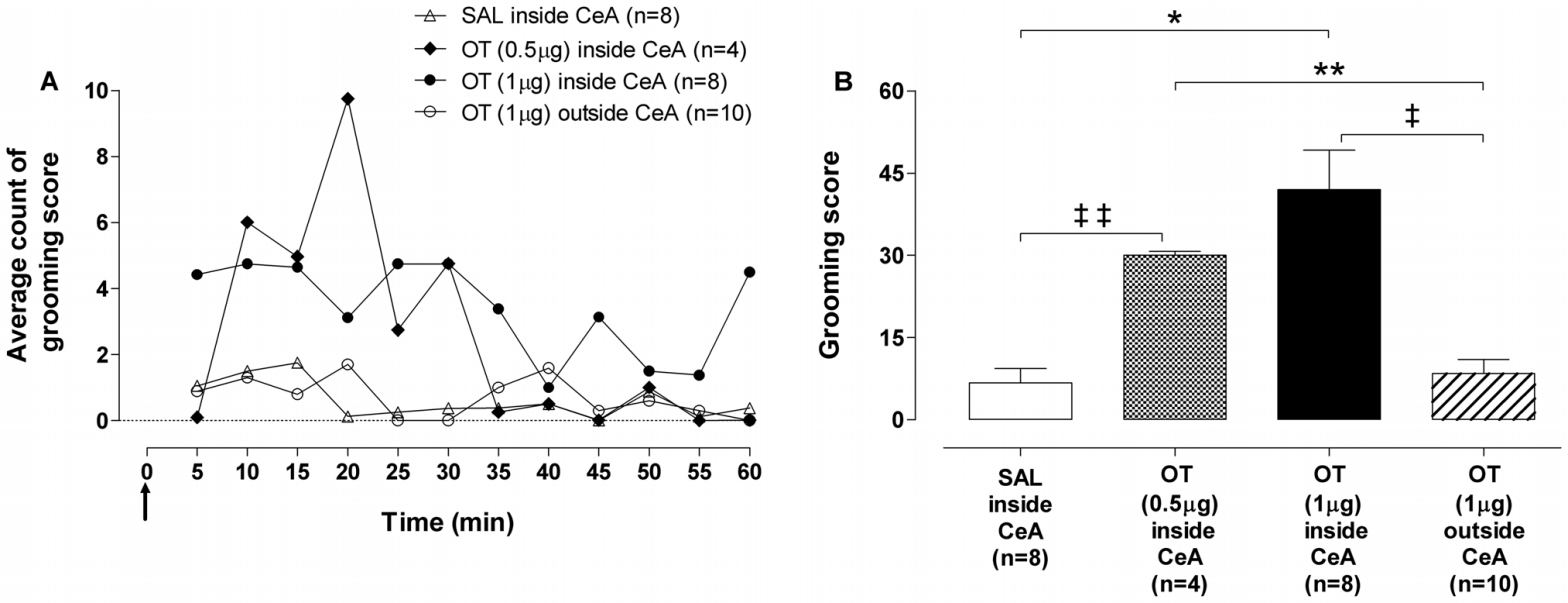
Grooming score following oxytocin microinjection into the central nucleus of amygdala. (A) Grooming score average count showing 5-min behavior sampling curve of oxytocin (OT) 0.5 µg, OT 1 µg or saline (SAL) into the central nucleus of amygdala (CeA) group and OT 1 µg outside CeA group. (B) grooming score (sum of grooming behavior over 60 min) of OT 0.5 µg (checkered bar), OT 1 µg (black bar, n = 8) or SAL (open bar) inside CeA group and OT 1 µg outside CeA group (striped bar). The arrow indicates the moment of microinjections. Data shown represent the means. (‡ ‡) SAL inside CeA group *vs* OT 0.5 µg inside CeA group; (*) SAL inside CeA group *vs* OT 1 µg inside CeA group; (**) OT 0.5 µg inside CeA group *vs* OT 1 µg outside CeA group; (‡) OT 1 µg inside CeA group *vs* OT 1 µg outside CeA group. p<0.05, *Poisson* model.

Bilateral microinjections of OT (0.5 µg or 1 µg) into CeA increased grooming score in a dose dependent manner (Figure 1B). We observed that the grooming score obtained after microinjections of OT (0.5 µg or 1 µg) into CeA was greater than following injection of SAL into CeA or OT (1 µg) outside CeA (Figure 1B). Figure 1A summarizes the grooming score obtained in 1 h showing that the frequencies of grooming were lower in the SAL inside CeA and OT (1 µg) outside CeA groups, when compared to the OT (0.5 µg or 1 µg) inside CeA group. Grooming frequencies were higher in the group OT (0.5 µg) inside CeA than in SAL inside CeA at 0–30 and 50–60 minutes. Grooming frequencies were higher in the group OT (1 µg) inside CeA than in SAL inside CeA at 0–35, 40–45 and 50–60 minutes (Figure 1A). Higher frequencies of grooming were observed in the group OT (1 µg) inside CeA when compared to OT (1 µg) outside CeA at 0–15, 20–35, 40–45 and 50–60 minutes (Figure 1A). Grooming frequencies were higher in the group SAL inside CeA than in OT (1 µg) outside CeA group at 15–30, 35–45 and 55–60 (Figure 1A).

### Arterial pressure and heart rate

**Figure 2:**
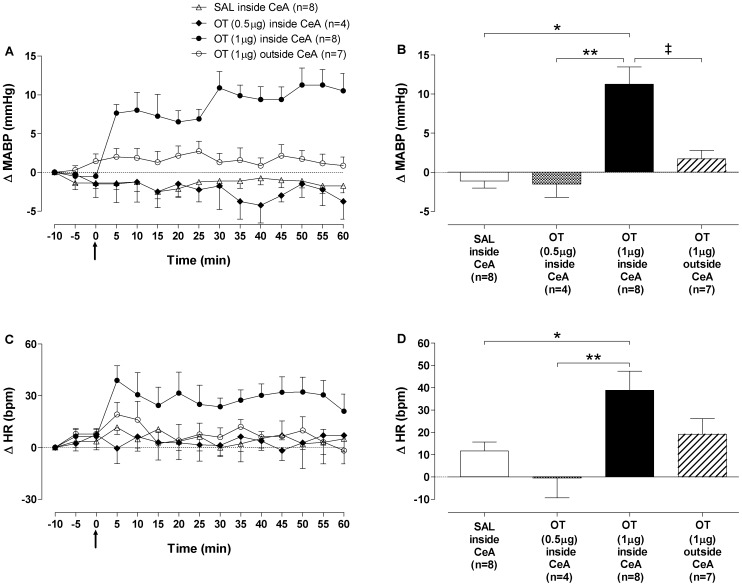
Hemodynamic responses to oxytocin microinjection into the central nucleus of amygdala. (A) Time-course of mean arterial blood pressure (ΔMABP, mmHg) and (C) heart rate (ΔHR, bpm) changes for 60 min after bilateral microinjections of oxytocin (OT) 0.5 µg, OT 1 µg or saline (SAL) into the central nucleus of amygdala (CeA) group and OT 1 µg outside CeA group. (B) Maximum responses in mean arterial blood pressure (ΔMABP, mmHg) at 50 min and (D) heart rate (ΔHR, bpm) at 5 min after bilateral microinjections of OT 0.5 µg (black-white bar), OT 1 µg (black bar) or SAL (open bar) inside CeA group and OT 1 µg outside CeA group (striped bar). The arrow indicates the moment of microinjections. Data presented are the means ± standard error of the mean. (*) SAL inside CeA group *vs* OT 1 µg inside CeA group; (**) OT 0.5 µg inside CeA group *vs* OT 1 µg inside CeA group; (‡) OT 1 µg inside CeA group *vs* OT 1 µg outside CeA group. p<0.001, Two-way ANOVA followed by Bonferroni's *post hoc* test.

No differences were observed in baseline MABP and HR among the groups. Figure S2 (Text S1) shows tracings from a representative rat and illustrates the changes in MABP and HR after microinjections of OT (1 µg) into the CeA. Figures 2A–2B summarize the data showing an increase in MABP, which began at 5 min and reached the maximum response at 50 min after microinjections. The pressor response induced by microinjection of OT (1 µg) into the CeA was associated with a significant increase in HR, which reached the maximum response 5 min after the microinjections (Figures 2C–2D). The pressor and tachycardic responses induced by OT (1 µg) were observed during 60 min after microinjections. Analysis of cardiovascular parameters 24 h after the microinjections showed that the effects of OT (1 µg) were reversible, because BP and HR returned to the baseline values (data not shown). In the SAL+OT group, cardiovascular parameters were similar to control levels 90 min following OT microinjections (data not shown). Lastly, changes in cardiovascular parameters following bilateral microinjections of SAL into CeA, OT (0.5 µg) into CeA or OT (1 µg) outside CeA were found lower when compared to those observed in OT (1 µg) inside the CeA group (Figure 2).

### Systolic arterial pressure and pulse interval variability

**Figure 3:**
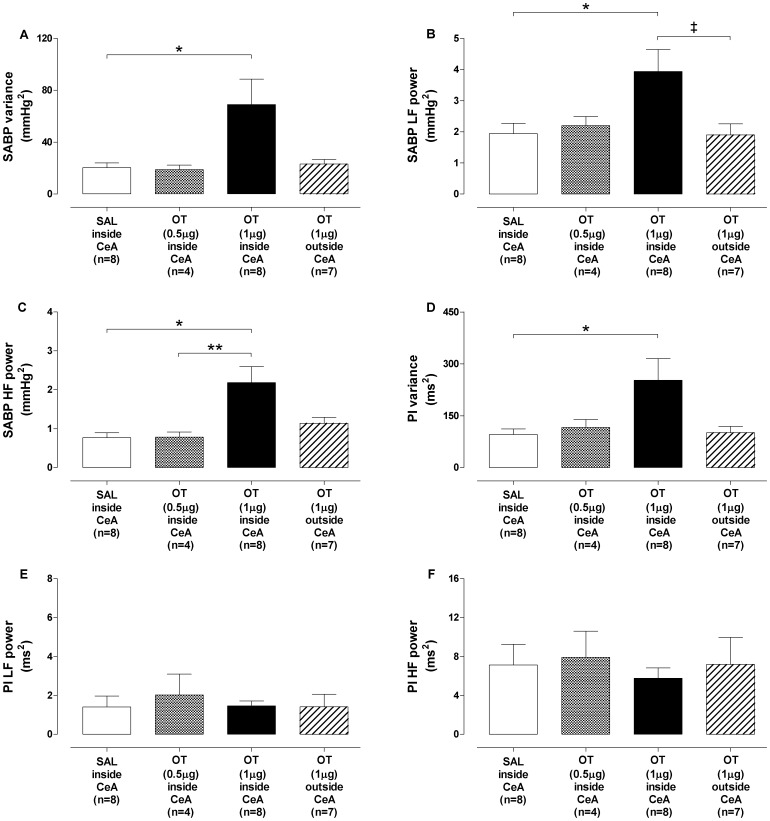
Systolic arterial blood pressure and pulse interval variability following oxytocin microinjection into central nucleus of amygdala. (A–C) Systolic arterial blood pressure (SABP) and (D–F) pulse interval (PI) variability analysis at 10 min after bilateral microinjections of oxytocin (OT) (black-white bar, n = 4), OT 1 µg (black bar, n = 8) or SAL (open bar, n = 8) into the central nucleus of amygdala (CeA) group and OT 1 µg outside CeA group (striped bar, n = 7). A and D: variance (σ^2^); B, C, E and F: power of the low and high frequency (LF; HF) bands. Data shown represent the means ± standard error of mean. (*) SAL inside CeA group *vs* OT 1 µg inside CeA group; (**) OT 0.5 µg inside CeA group *vs* OT 1 µg inside CeA group; (‡) OT 1 µg inside CeA group *vs* OT 1 µg outside CeA group.p<0.05, One-way ANOVA followed by Bonferroni's *post hoc* test.

The results of systolic arterial blood pressure (SABP) and pulse interval (PI) variability analysis at 10 min after microinjections of OT (0.5 µg or 1 µg) or SAL are shown in Figure 3. The OT (1 µg) into the CeA group exhibited higher SABP variance as compared to SAL into CeA group (Figure 3A). This was associated with an increase in LF (Figure 3B) and HF (Figure 3C) power of the SABP spectrum, which started at 10 min and remained elevated by the end of the 60 min observation period. The group OT (1 µg) inside CeA also exhibited higher PI variance in relation to the SAL inside CeA group (Figure 3D). However, no changes were observed in the power of the LF and HF bands of the PI spectrum (Figures 3E–3F) among the groups. SABP and PI variability analysis 24 h after the microinjections showed that the effects of OT (1 µg) into CeA were reversible (data not shown). Microinjections of OT (0.5 µg) into CeA or OT (1 µg) outside CeA produced no change in the SABP and PI variability, when compared to SAL inside CeA group (Figure 3).

### Spontaneous baroreflex sensitivity

No differences were observed on the number of UP and DOWN baroreflex sequences among the groups microinjected with OT (0.5 µg or 1 µg) or SAL into the CeA (Figure S3A; Supporting Information). However, BRS was reduced in the OT (1 µg) inside the CeA group, when compared to SAL inside CeA group (Figures S3B–S3D). Microinjections of OT (0.5 µg) into CeA or OT (1 µg) outside of the CeA produced no change in the BRS, when compared with SAL inside CeA group (Figures S3B–S3D). Analysis of BRS 24 h after the microinjections showed that the effects of OT (1 µg) were reversible (data not shown).

### Ventilation

**Figure 4:**
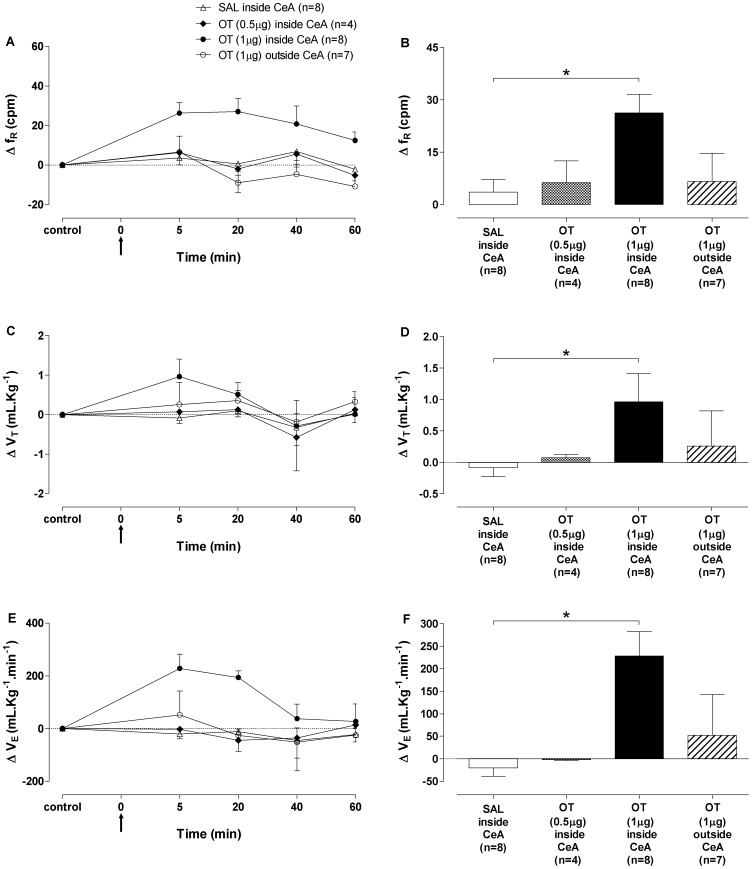
Respiratory responses to oxytocin microinjection into the central nucleus of amygdala. (A) Time-course of respiratory frequency (Δf_R_, cpm), (C) tidal volume (ΔV_T_, mL.Kg^−1^) and (E) minute ventilation (ΔV_E_, mL.Kg^−1^.min^−1^) changes for 60 min after bilateral microinjections of oxytocin (OT) 0.5 µg, OT 1 µg or saline (SAL) into the central nucleus of amygdala (CeA) group and OT 1 µg outside CeA group. Maximum responses in (B) respiratory frequency (Δf_R_, cpm), (D) tidal volume (ΔV_T_, mL.Kg^−1^) and (F) minute ventilation (ΔV_E_, mL.Kg^−1^.min^−1^) 5 min after bilateral microinjections of OT 0.5 µg (black-white bar), OT 1 µg (black bar) or SAL (open bar) inside CeA group and OT 1 µg outside CeA group (striped bar). The arrow indicates the moment of microinjections. Data shown represent the means ± standard error of mean. (*) SAL inside CeA group *vs* OT 1 µg inside CeA group; p<0.05, Two-way ANOVA followed by Bonferroni's *post hoc* test.

No differences were observed on f_R_, V_T_ and V_E_ among the groups. Microinjections of OT (1 µg) into the CeA produced increased f_R_ (Figures 4A–4B), V_T_ (Figures 4C–4D) and V_E_ (Figures 4E–4F), which reached maximum response at 5 min after microinjections. The ventilatory responses produced by OT (1 µg) were reversed to baseline values after 40 min. Microinjections of OT (0.5 µg) into CeA or OT (1 µg) outside CeA produced no change in the respiratory parameters, when compared with SAL inside CeA group (Figure 4).

### Oxytocin receptor antagonism

**Figure 5:**
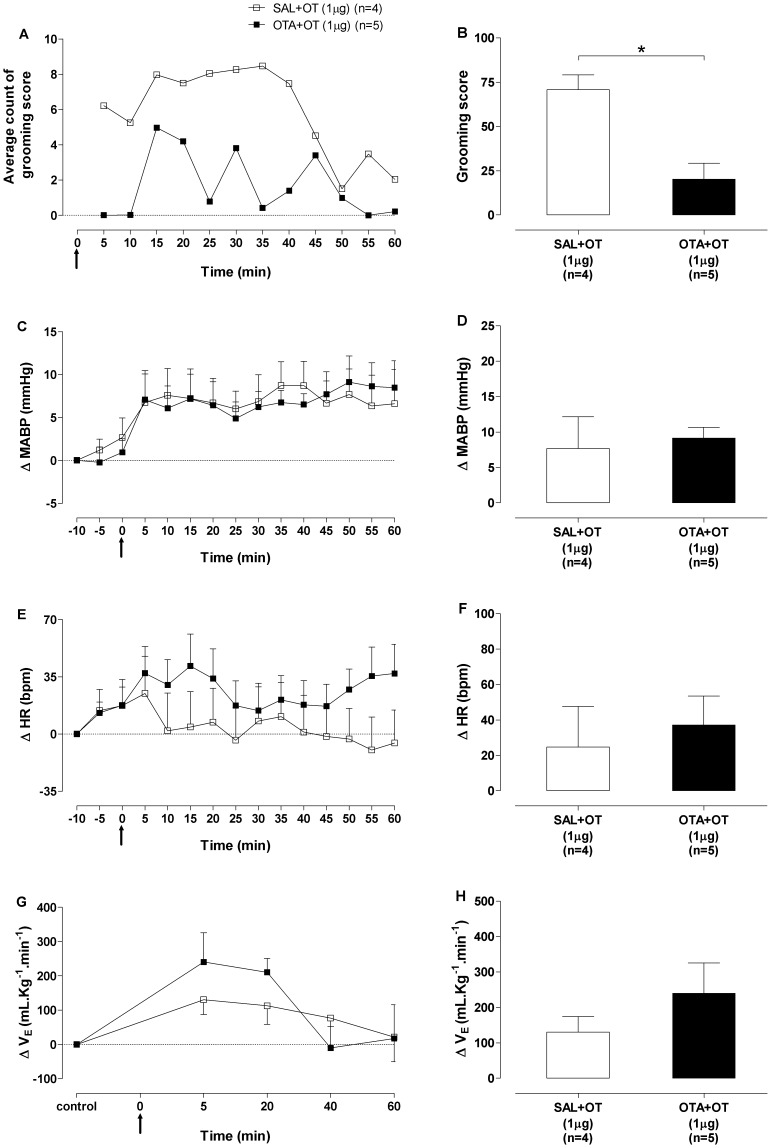
Behavioral, hemodynamic and respiratory responses to oxytocin antagonist. (A) Grooming score average count of the 5-min behavior sampling window of the saline+oxytocin (SAL+OT) 1 µg and vasotocin+OT (OTA+OT) 1 µg groups. (B) Grooming score (mean of 60 min) of the SAL+OT 1 µg (open bar) and OTA+OT 1 µg (black bar) groups. Time-course of (C) mean arterial blood pressure (ΔMABP, mmHg), (E) heart rate (ΔHR, bpm) and (G) minute ventilation (ΔV_E_, mL.Kg^−1^.min^−1^) changes for 60 min after bilateral microinjections of OT 1 µg into the central nucleus of amygdala (CeA) in the SAL+OT 1 µg and OTA+OT 1 µg groups. Maximum responses in (D) mean arterial blood pressure (ΔMABP, mmHg) at 50 min, (F) heart rate (ΔHR, bpm) at 5 min and (H) minute ventilation (ΔV_E_, mL.Kg^−1^.min^−1^) at 5 min after bilateral microinjections of OT 1 µg into the CeA in the SAL+OT 1 µg (open bars) and OTA+OT 1 µg (black bars) groups. The arrow indicates the moment of microinjections. Data shown represent the means ± standard error of mean. (*) SAL+OT 1 µg group *vs* VASO+OT 1 µg group; p<0.05, *Poisson* model.

In agreement with previous findings of our laboratory ^5^, pretreatment with OTA (1 µg/200 nL) into the CeA attenuated the hypergrooming induced by OT (Figures 5A–5B). In contrast, OTA pretreatment did not affect the increase in MABP (Figures 5C–5D), HR (Figures 5E–5F) and V_E_ (Figures 5G–5H) induced by microinjections of OT (1 µg) into the CeA. Likewise, no differences were observed in SABP variability, PI variability and BRS of rats microinjected with OTA before OT, in relation to rats microinjected with SAL. In addition, microinjections of OTA into the CeA of rats did not affect the cardiovascular parameters. In these experiments, MABP and HR values were continuously assessed until the reversal of cardiovascular responses induced by SAL+OT or OTA+OT, which occurred 90 min after OT microinjections.

The behavioral sequences of the SAL+OT and OTA+OT groups are shown in Figure S4, (Text S1).

## Discussion

Our results show for the first time that hypergrooming evoked by bilateral microinjections of OT (1 µg) into the CeA of rats is accompanied by marked alterations in cardiovascular and ventilatory parameters. We observed that grooming behavior, MABP, HR and minute ventilation of animals become elevated 5 min after microinjections of OT (1 µg) into the CeA. In addition, rats showed increased LF and HF power of the SABP spectrum, indexes associated with vascular sympathetic modulation and respiration modulation, respectively. Elevation of the MABP, HR, LF and HF power persisted throughout the entire 90 min observation period. Rats that received OT (1 µg) into the CeA also exhibited reduced BRS and increased V_E_ while animals that received microinjections of OT (1 µg) outside CeA, OT (0.5 µg) or SAL into CeA did not exhibit changes on these parameters.

The role of OT in the central regulation of the cardiovascular system was previously investigated but the results were inconclusive [28]–[34]. In some studies intraventricular (icv) or intracisternal administration of OT elicited pressor or tachycardic responses [30], [33] whereas in others icv infusion of OT for 5 days elicited a hypotensive response [32]. It has also been shown that OT reduces HR acceleration during exercise [31]. In spite of the fact that previous studies demonstrated that central administration of OT is followed by important cardiovascular alterations, the interaction of these physiological changes with behavioral disturbances, including compulsive behavior, remain to be elucidated.

Then, we evaluated the simultaneous behavioral and cardiorespiratory responses to OT into the CeA, an experimental model of compulsion [5]. Our data show that central administration of increasing doses of OT (0.5 or 1 µg) into the CeA produced hypergrooming in a dose dependent manner. This pattern of dose-response curve is similar to that found for hypergrooming induced by icv injection of OT in rats [3]. It is important to note that only the dose of OT of 1 µg was effective to increase BP and HR during hypergrooming. Although these behavioral and cardiovascular responses induced by OT (1 µg) began 5 min after microinjections, they were not always synchronous. We observed that at times 35–40 and 45–55 min after microinjections of OT (1 µg) into the CeA, rats did not present hypergrooming, while the BP reached a peak response 50 min after microinjections and was still elevated 90 min following microinjections.

OT receptors are metabotropic G-protein coupled receptors, coupled to both Gq and Gi, which has been identified in a large variety of cells, including neurons [35], [36]. Yoshimura et al. [37] identified OT receptor RNA in large quantities in central and medial amygdala. In rats and other rodents, this region shows large densities of OT receptors, as revealed by autoradiography [36], [38], [39]. In order to evaluate if the behavior and cardiorespiratory effects induced by OT microinjection into the CeA was OT-selective and did not activate other system receptors, such as vasopressin [40], we used the specific OT receptor antagonist OTA [22]. Our data show that OTA pretreatment (1 µg) attenuated the occurrence of OT-induced hypergrooming, indicating that this behavioral effect is mediated by a direct activation of OT receptors. In contrast, the cardiorespiratory responses to OT were not affected by OTA pretreatment, indicating that different neural pathways and possibly independent neurochemical mechanisms mediate the behavioral and cardiorespiratory responses to OT in the CeA, and these responses are not affected by OTA pretreatment. Similar findings have been previously reported for CeA regulation of fear response, where it has been shown that OT gate cardiovascular and behavioral parameters through at least two distinct neural pathways [41]. Consequently, we think that the dissociation between the behavioral and cardiovascular responses associated to CeA OT-induced grooming may involve central actions of OT.

To our knowledge, our results represent the first demonstration of this phenomenon in the control of compulsive behavior. In our study, cardiac autonomic modulation was evaluated by means of SABP and PI variability analysis (i.e. non-invasive approach). We observed that the sustained increase in BP produced by OT (1 µg) was associated with increased LF power of the SABP spectrum, which in turn is a reliable marker for sympathetic modulation of vasomotor activity [42], [43]. For example, the loss of central sympathetic modulation can cause the absence of LF power [44], whereas stimulation of brainstem vasomotor center can linearly increase LF power [42]. Thus, we suggest that the pressor response induced by OT (1 µg) into CeA is, at least in part, due to an increase in vascular sympathetic modulation. On the other hand, LF and HF power of the PI spectrum, which are indexes for cardiac sympathetic [45] and vagal modulation [46], respectively, were not affected by OT (0.5 µg or 1 µg) into the CeA.

We observed that the pressor and tachycardic responses induced by OT (1 µg) into the CeA were associated with a reduction in BRS. The baroreflex is a negative feedback loop that controls BP by modulating the sympathetic and parasympathetic activity [47]. Anatomical and physiological studies showed that the CeA sends GABAergic projections to the nucleus tractussolitarii (NTS) [48], the primary termination site of vagal and glossopharyngeal afferent fibers in the brainstem [49]–[53]. These projections may provide an anatomical substrate for some modalities of functional inhibition of lower brainstem visceral reflexes, including the baroreflex [10]. Through this mechanism, the CeA may participate in cardiovascular regulatory events related to emotional behavior and defensive reaction [10]. Considering our findings we suggest that the increase in BP, HR and vascular sympathetic modulation induced by OT into the CeA is, at least partially, due to the reduction in BRS. In addition to governing the activity of the autonomic nervous system, baroreceptors also tonically inhibit the release of arginine vasopressin from the posterior pituitary [54]–[57]. Therefore, reduction in the BRS might lead to increased release of arginine vasopressin and induce hypertension [58]. This hypothesis would explain the sustained pressor response at 60 min even in the absence of grooming behavior.

A quite recent study [59] characterized the enhancement of blood pressure and heart rate in air-jet stressed rats. In brief, using a clever set of experiments the authors show first, that air-jet stress induces blood pressure and heart rate increases in both Wistar Kyoto (WKY) and spontaneous hypertensive rats (SHR) rats. Secondly, they show that i.c.v. OT potentiated the increased pressor responses in stressed WKY rats, effect that was antagonized by OT receptor antagonist (OTA). By contrast, OT blocked the stress-dependent blood pressure increase in SHR animals. Finally, when the animals of both groups WKY were treated i.c.v. with vasopressin V1aR antagonists, the enhanced stress-dependent pressor response to OT was blocked. Therefore, further experiments will be done with the WAR strain and their Wistar controls, in order to verify if, as in the case of the WKY and the SHR animals, a potential dissociation or convergence of effects can be elucidated with central pre-treatments with vasopressin V1aR antagonists.

Another hypothesis for this uncoupling between cardiovascular and behavioral responses to OT, as evidenced by OTA pretreatment, is that the neural processing underlying each response may be fundamentally different. Compulsive behavior induced by OT into the CeA is most likely caused and maintained due to a sustained activation loop between the CeA and the hypothalamic grooming area mediated by recurrent OT connections [5]. Therefore, the intensity of hypergrooming response would be directly modulated by the amount of OT receptors in the CeA available for binding, which would explain why antagonism of these receptors with OTA decreased the hypergrooming response. The cardiovascular response to OT is probably mediated by a downstream effect of CeA output activity to the brainstem, primarily through inhibitory GABAergic connections to the NTS [48]. In this context, OT receptor stimulation in the CeA would only provide an initial trigger for downstream events that ultimately leads to a sustained reduction in BRS, and the intensity of cardiovascular responses would be independent of recurrent OT receptor stimulation in CeA, as long as there is an initial OT stimulus. This would explain why OTA in the CeA partially inhibits the hypergrooming responses to OT microinjection but does not change the cardiovascular responses.

It must be emphasized that the cardiovascular responses induced by OT (1 µg) were accompanied by changes in ventilation. Conversely, these rats displayed increased HF power of SABP spectrum, an index for respiratory modulation. Similarly to the pressor response, the ventilatory responses induced by OT (1 µg) were not synchronous with hypergrooming, because the increase in ventilation reached a peak at 5 min, reverting to control levels 40 min after microinjections. Neuroanatomical studies have demonstrated that the CeA sends projections to different areas involved in respiratory control, including the NTS [49]–[53] and the parabrachial nucleus [60]. Functional studies showed that CeA plays an important role in respiratory control. Nie and Liu [14] observed that activation of CeA neurons of anesthetized rabbits increased ventilation, suggesting that this nucleus may play an important role in facilitating the basic respiratory rhythm. Thus, it is plausible to propose that ventilatory responses induced by OT may be due to a modulation of brainstem respiratory networks, which are functionally coupled to pre-ganglionic sympathetic neurons [61]–[63]. Therefore, the cardiovascular and ventilatory components activated during hypergrooming induced by OT (1 µg) into CeA may involve functionally diverse neurons and different central pathways.

Several studies have indicated that grooming behaviors are mainly induced in anxiogenic and conflict situations [2]. Although OT seem to represent counter mechanisms to block or alleviate anxiety [19], [64], we observed that the cardiorespiratory responses induced by OT into CeA were similar to those experienced during flight, fear and anxiety [12], [15]. Kalueff and Tuohimaa [65] suggested that some grooming patterns can be used as behavioral markers of stress in rats. Then, it is possible to speculate that the behavioral patterns observed in OT-induced hypergrooming may be stressful for the animal, inducing cardiorespiratory alterations.

Patients with behavioral disorders show higher incidence of cardiovascular diseases [16]–[18] but this fact is usually discussed as an indirect consequence of stress and destructive behavioral patterns characteristic of each psychiatric condition [16]. We hypothesize that the behavioral symptoms and cardiovascular co-morbidities of obsessive-compulsive disorders might share common pathophysiological origins, including a counter-adaptive amygdala OT-dependent mechanism.

In summary, our findings demonstrate that administration of OT (1 µg) into the CeA not only induces hypergrooming but also affects cardiorespiratory parameters. These behavioral and cardiorespiratory responses to OT in the CeA seems be mediated by different neural pathways and may involve distinct neurochemical mechanisms. The increase in BP is associated with a facilitation of vascular sympathetic outflow and reduction in BRS. These OT-induced responses seem to involve the activation of specific CeA neurons. Our results may shine a new light on how we understand the pathophysiology of obsessive-compulsive disorders.

Then, additional experimental research is needed to further elucidate the neurohumoral mechanisms involved on this behavioral-cardiorespiratory relationship, as well as the possible influence of this system in the pathogenesis of cardiorespiratory maladies in patients suffering from obsessive-compulsive disorders. Whether or not cardiorespiratory dysfunctions (e.g. autonomic imbalance and changes in f_R_) can be used as a fingerprint of compulsive behavior is another important issue that requires further investigation. A recent study by Pittig et al [66] demonstrates that reduced HF power of the HR spectrum can be used as an index for inhibitory deficits in patients with panic disorders, generalized anxiety and social anxiety, which is in line with the so-called autonomic inflexibility in patients with these anxiety disorders. However, elevated HR responses to hyperventilation seems to be specific of panic disorder and generalized anxiety, as the authors did not observe significant autonomic variations in obsessive-compulsive disorder (OCD) patients. However, the fact that the subjects were receiving drug treatment was a confounding factor that may have obscured the autonomic symptoms of the disease. These limitations of clinical research highlight the importance of applying experimental, highly controlled, animal models of neuropsychiatric disorders, such as the one used in the current study (i.e. OT-mediated modulation of compulsive behavior, and cardiorespiratory responses), to characterize the association between behavioral and autonomic manifestations of neuropsyquiatric disorders.

## Supporting Information

Figure S1 **Microinjections placement.** (A) Photomicrograph of a coronal section of the brain of one rat showing the bilateral microinjection sites located in the central nucleus of amygdala (CeA; head of arrows). (B) Diagrammatic representation of a transverse section of the brain (-2.3 mm caudal to the bregma) based on the atlas of Paxinos and Watson [1] indicating the center of bilateral microinjections of oxytocin (OT) 0.5 µg (▴), OT 1 µg (○) and saline (SAL; Δ) into the CeA of 20 rats with positive histology and the sites of misplaced microinjections of OT 1 µg outside CeA of 7 rats (•). Panel C is a diagrammatic representation of the same transverse section of the brain indicating the center of bilateral microinjections of SAL+OT 1 µg (□) and vasotocin+OT (OTA+OT) 1 µg (▪) into the CeA of 20 rats with positive histology and the sites of misplaced microinjections of SAL+OT 1 µg (◊) and OTA+OT (♦) outside CeA of 7 rats. MGP, medial globuspallidus; CPU, caudate putamen; IC, internal capsule. The calibration bar corresponds to 5 mm.
(TIF)

Figure S2 **Typical hemodynamic responses to oxytocin microinjection into the central nucleus of amygdala.** Typical tracings of one rat representative of the group illustrating the changes in pulsatile arterial pressure (PAP, mmHg), mean arterial blood pressure (MABP, mmHg) and heart rate (HR, bpm), 10 min before (control) and 60 min after bilateral microinjections of OT (1 µg) into the central nucleus of amygdala (CeA). The arrow indicates the moment of microinjections.
(TIF)

Figure S3 **Baroreflex responses to oxytocin microinjection into the central nucleus of amygdala.** Number of UP and DOWN spontaneous baroreflex sequences detected in 10,000 beats (A), slopes of UP sequences (B), slopes of DOWN sequences (C), slope of all sequences (D) of oxytocin (OT) 0.5 µg (black-white bar), OT 1 µg (black bar) or SAL (open bar) into the central nucleus of amygdala (CeA) group and OT 1 µg outside CeA group (striped bar). Data presented are the means ± standard error of mean. (*) SAL inside CeA group *vs* OT 1 µg inside CeA group. p<0.05, One-way ANOVA followed by Bonferroni's *post hoc* test.
(TIF)

Figure S4 **Neuroethological analysis of the behavioral sequences associated with oxytocin microinjection into central nucleus of amygdala.** (A) Flowchart calibration: the height of the rectangles represents the frequency of a behavioral item and the length corresponds to the duration of each behavior during the observation windows; arrows represent statistical values (X^2^> 3.84; p < 0.05) highlighting the strength of association between pairs of behaviors (dyads). The major behavioral clusters are highlighted by color. The use of colors and circles and the calibration of rectangles in the flowcharts are for illustration purposes and do not have any impact in the statistical analysis. Neuroethological evaluation of behavioral sequences after saline+oxytocin (SAL+OT) or vasotocin+OT (OTA+OT) bilateral microinjections in the central nucleus of amygdala (CeA) in four periods of 5 minutes observation windows. (B) Wistar SAL+OT Group. (C) Wistar OTA+OT Group. See complete description in the text. Orofacial automatisms: MT - Mastigatory; WDS - Wet Dog Shaking; YA – Yawn. Other behaviors: ER - Erect Posture; IM – Immobility; Movements; SCA - Scanning; SN - Sniffing; WA - Walking:; Grooming behavioral items: GRR -Grooming of body (right); GRL - Grooming of body (left); GRG - Grooming of genitalia; GRH - Grooming of head; LIC - Licking of claws; LCR1 - Licking of claws (right, anterior); LCR2 - Licking of claws (right, posterior); LCL1 - Licking of claws (left, anterior); LCL2 - Licking of claws (left, posterior); GRF - Grooming of face; SCRL –scratch left, SCRR –scratch right.
(TIF)

Text S1 **Methods, Results and References.**
(DOCX)
