# Supplementary figures and images for: Behavioral and Cardiorespiratory Responses to Bilateral Microinjections of Oxytocin into the Central Nucleus of Amygdala of Wistar Rats, an Experimental Model of Compulsion
Source: PLoS One. 2014 Jul 18;9(7):e99284. doi: 10.1371/journal.pone.0099284 (PMC4103777; doi:10.1371/journal.pone.0099284)

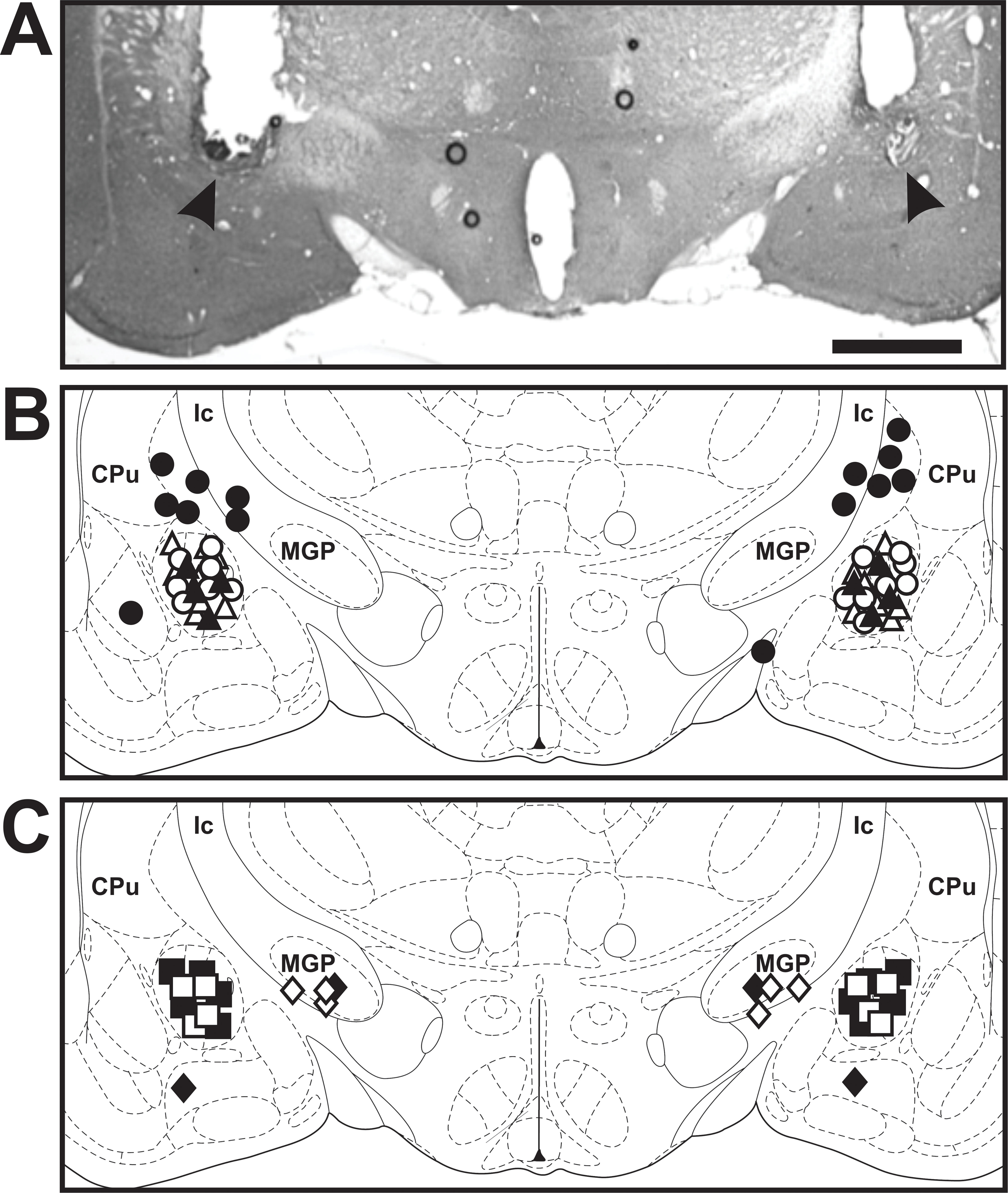

Supplement: Figure S1 — Microinjections placement. (A) Photomicrograph of a coronal section of the brain of one rat showing the bilateral microinjection sites located in the central nucleus of amygdala (CeA; head of arrows). (B) Diagrammatic representation of a transverse section of the brain (-2.3 mm caudal to the bregma) based on the atlas of Paxinos and Watson [1] indicating the center of bilateral microinjections of oxytocin (OT) 0.5 µg (▴), OT 1 µg (○) and saline (SAL; Δ) into the CeA of 20 rats with positive histology and the sites of misplaced microinjections of OT 1 µg outside CeA of 7 rats (•). Panel C is a diagrammatic representation of the same transverse section of the brain indicating the center of bilateral microinjections of SAL+OT 1 µg (□) and vasotocin+OT (OTA+OT) 1 µg (▪) into the CeA of 20 rats with positive histology and the sites of misplaced microinjections of SAL+OT 1 µg (◊) and OTA+OT (♦) outside CeA of 7 rats. MGP, medial globuspallidus; CPU, caudate putamen; IC, internal capsule. The calibration bar corresponds to 5 mm. (TIF) [file pone.0099284.s001.tif]

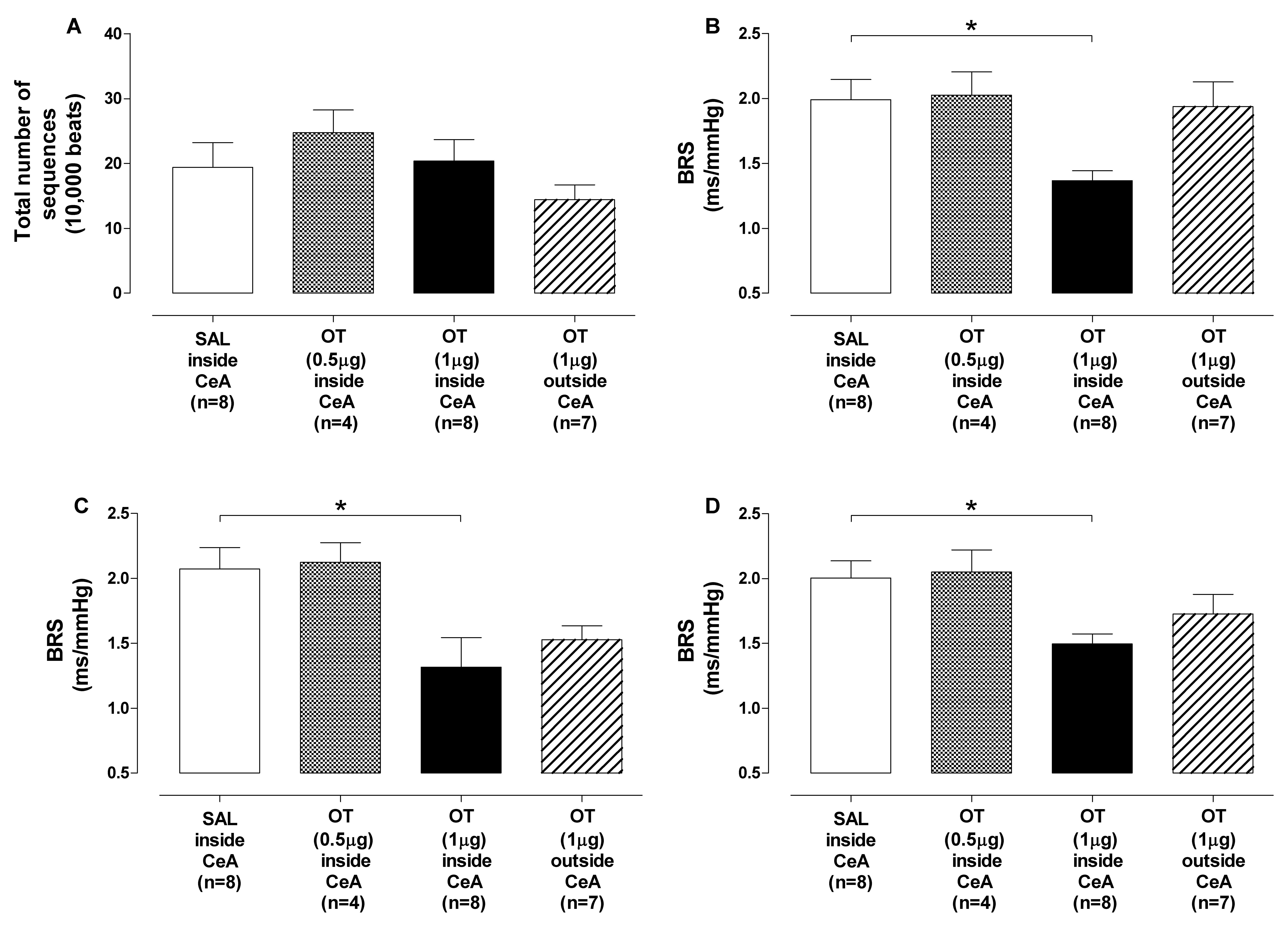

Supplement: Figure S3 — Baroreflex responses to oxytocin microinjection into the central nucleus of amygdala. Number of UP and DOWN spontaneous baroreflex sequences detected in 10,000 beats (A), slopes of UP sequences (B), slopes of DOWN sequences (C), slope of all sequences (D) of oxytocin (OT) 0.5 µg (black-white bar), OT 1 µg (black bar) or SAL (open bar) into the central nucleus of amygdala (CeA) group and OT 1 µg outside CeA group (striped bar). Data presented are the means ± standard error of mean. (*) SAL inside CeA group vs OT 1 µg inside CeA group. p<0.05, One-way ANOVA followed by Bonferroni's post hoc test. (TIF) [file pone.0099284.s003.tif]

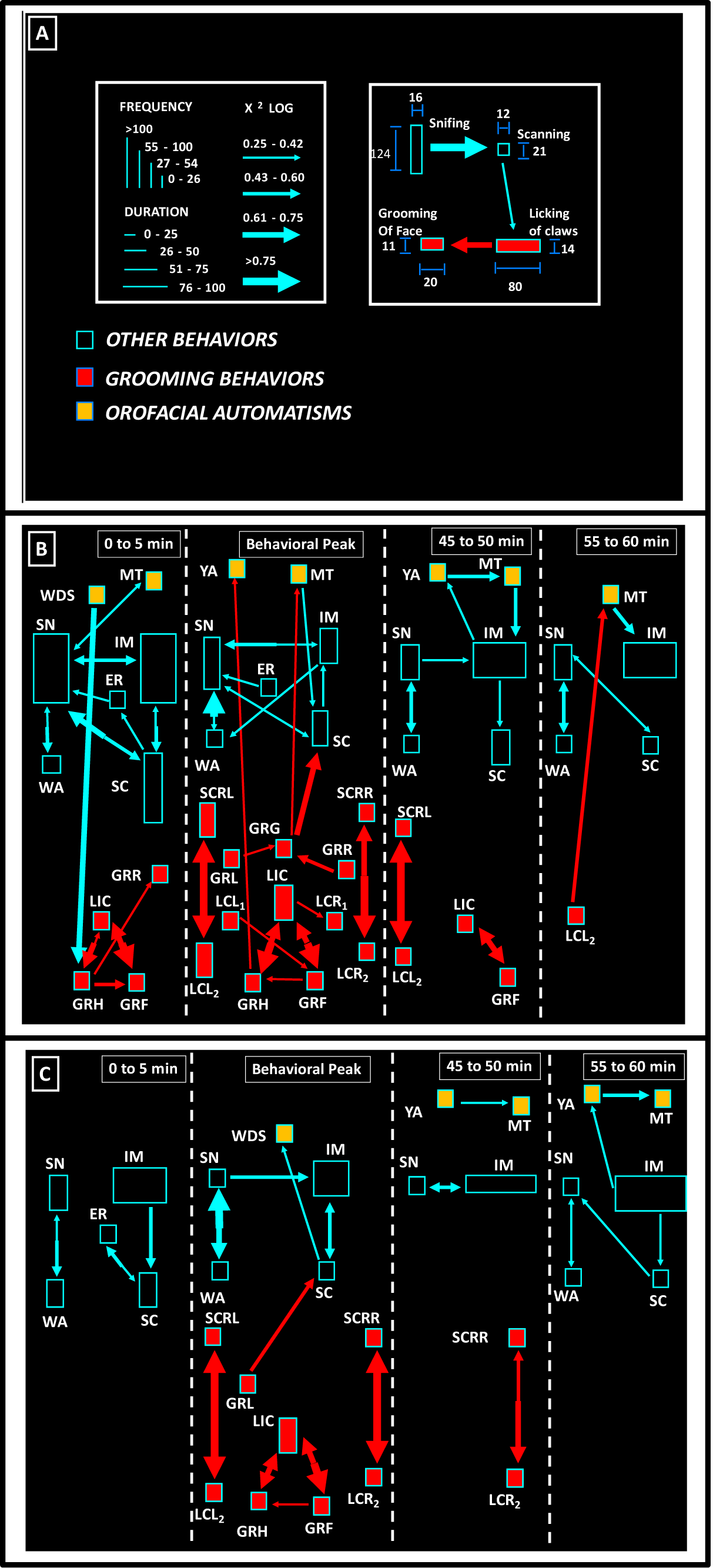

Supplement: Figure S4 — Neuroethological analysis of the behavioral sequences associated with oxytocin microinjection into central nucleus of amygdala. (A) Flowchart calibration: the height of the rectangles represents the frequency of a behavioral item and the length corresponds to the duration of each behavior during the observation windows; arrows represent statistical values (X2> 3.84; p < 0.05) highlighting the strength of association between pairs of behaviors (dyads). The major behavioral clusters are highlighted by color. The use of colors and circles and the calibration of rectangles in the flowcharts are for illustration purposes and do not have any impact in the statistical analysis. Neuroethological evaluation of behavioral sequences after saline+oxytocin (SAL+OT) or vasotocin+OT (OTA+OT) bilateral microinjections in the central nucleus of amygdala (CeA) in four periods of 5 minutes observation windows. (B) Wistar SAL+OT Group. (C) Wistar OTA+OT Group. See complete description in the text. Orofacial automatisms: MT - Mastigatory; WDS - Wet Dog Shaking; YA – Yawn. Other behaviors: ER - Erect Posture; IM – Immobility; Movements; SCA - Scanning; SN - Sniffing; WA - Walking:; Grooming behavioral items: GRR -Grooming of body (right); GRL - Grooming of body (left); GRG - Grooming of genitalia; GRH - Grooming of head; LIC - Licking of claws; LCR1 - Licking of claws (right, anterior); LCR2 - Licking of claws (right, posterior); LCL1 - Licking of claws (left, anterior); LCL2 - Licking of claws (left, posterior); GRF - Grooming of face; SCRL –scratch left, SCRR –scratch right. (TIF) [file pone.0099284.s004.tif]
