# Supplementary material for: Behavioral and Cardiorespiratory Responses to Bilateral Microinjections of Oxytocin into the Central Nucleus of Amygdala of Wistar Rats, an Experimental Model of Compulsion
Source: PLoS One. 2014 Jul 18;9(7):e99284. doi: 10.1371/journal.pone.0099284 (PMC4103777; doi:10.1371/journal.pone.0099284)
Supplement: Text S1 — Methods, Results and References. (DOCX) [file pone.0099284.s005.docx]

**Behavioral and cardiorespiratory responses to bilateral microinjections of oxytocin into the central nucleus of amygdala of Wistar rats, an experimental model of compulsion**

**SUPPORTING INFORMATION**

**Supporting Methods**

*Animals*

Animals were assigned to the following six groups: (I) rats that received bilateral microinjections of saline (SAL, vehicle) inside central nucleus of amygdala (CeA; n=8); (II) rats that received bilateral microinjections of oxytocin (OT; 0.5 μg; 0.5 nmol) inside CeA (n = 4); (III) rats that received bilateral microinjections of OT (1 μg; 1 nmol) inside CeA (n = 8); (IV) rats that received bilateral microinjections of OT (1 μg; 1 nmol outside CeA (n=7); (V) rats that received bilateral microinjections of saline followed by OT (1 μg; SAL+OT) inside CeA (n=4); and (VI) rats that received bilateral microinjections of the OT antagonist vasotocin (OTA, 1 μg/200 nL; 0.44 nmol; d(CH_2_)^1^_5_, Try(Me)^2^, Thr^4^, Orn^8^, Tyr-NH ^9^_2_; Bachem, Torrance, CA, USA) followed by OT (1 μg; OTA+OT) inside CeA (n=5).

*Implantation of guide cannulae and catheter*

With the purpose of implantation of guide cannulae, the skull was first leveled between the bregma and lambda. The coordinates of the CeA relative to the bregma suture were: antero-posterior, -2.0 mm; medio-lateral, ± 4.0 mm and dorso-ventral, -7.0 mm from the bone surface in accordance with the Paxinos and Watson rat brain atlas [1]. Next, the guide cannulae were fixed to the skull with methacrylate and watch screws and closed with a metallic obturator until the beginning of the experiments, when it was removed and replaced by a metallic needle (33 gauge, Small Parts, Miami Lakes, FL, USA) 1.0 mm longer than the guide cannulae used to perform the microinjections into the CeA. To avoid infections, each animal received prophylactic injections of veterinary pentabiotic for small mammals (0.1 mL/100g, FORT DODGE Saúde Animal LTDA, Campinas, SP) in the beginning of these surgical procedures.

The catheter was tunneled subcutaneously and exteriorized through the back of the neck to prevent them from being chewed. The rats were then housed singly and their respiratory movements were monitored until they regained consciousness. After 24 h, rats were allowed to adapt to the recording room and had the pulsatile arterial pressure recorded.

*Microinjections into the CeA of rats*

Microinjections into the CeA were performed using two needles (33-gauge, Small Parts, Miami Lakes, FL, USA), which were previously connected by PE-10 tubing to a 5 μL syringe (Hamilton, Reno, NV, USA). The needles and their respective syringes were linked to an infusion pump (Harvard Apparatus PHD 2000, Holliston, MA, USA), allowing the bilateral microinjections could be performed at the same time. After removal of the obturator, the needles were carefully inserted into the respective guide cannulae and small pieces of polyethylene, previously connected to the external part of the guide cannulae, were used to fix the needles while the microinjections were performed with the rat inside the whole-body plethysmographic chamber. In all animals, the needles were maintained 1 min inside the guide cannula after the end of the injection.

*Cardiovascular recordings and analysis*

Rats had the arterial catheter connected to a pressure transducer (MLT0380, ADInstruments, Bella Vista, NSW, Australia) and pulsatile arterial pressure signal was amplified (Bridge Amp, ML221, ADInstruments) and recorded in conscious freely moving rats using an IBM/PC equipped with an analog-to-digital interface (1 kHz; PowerLab 4/25, ML845, ADInstruments). Next, recordings were processed by a computer software (LabChart v7.0, ADInstruments), which detects cycle-to-cycle inflection points in the signal, measures the time interval between adjacent arterial pressure pulses and generates beat-by-beat time series of systolic arterial blood pressure (SABP), mean arterial blood pressure (MABP), heart rate (HR) and pulse interval (PI).

Beat-by-beat time series of SABP and PI were used for cardiovascular variability analysis in the time and frequency domain. The overall variability of these series was calculated in the time domain and expressed as the variance (σ^2^) of the entire SABP and PI time series. We used freely available software (CardioSeries v. 1.0; http://www.danielpenteado.com) to perform SABP and PI variability analysis in the frequency domain, using Fast Fourier Transform. This analysis requires data collection at equal time intervals. Therefore, the beat-by-beat time series were converted to data points every 100 ms using a cubic spline interpolation (10 Hz). The interpolated series was divided into half-overlapping sequential sets of 512 data points (51.2 s). Before calculation of the power spectral density (PSD), a well-experienced researcher visually inspected the segments of interpolated time series searching for transients that could affect the PSD calculation. In order to certify the reliability of visual inspection, a Hanning window was used to attenuate side effects and all segments had the spectrum calculated using a direct Fast Fourier Transform algorithm for discrete time series. Next, all segments were visually inspected for abnormal spectra. All non-stationary data was excluded from the PSD calculations. The spectra were integrated (i.e. area under the curve calculation) in low-frequency (LF; 0.2-0.75 Hz) and high-frequency (HF; 0.75-3 Hz) bands [2,3] and results are expressed in absolute units (ms^2^ or mmHg^2^).

Baroreflex sensitivity (BRS) was evaluated in the time-domain by means of the sequence technique, originally described by Di Rienzo et. al. [4]. A freely available software (HemoLab v. 5.9; http://www.haraldstauss.com/HemoLab/HemoLab.html) was used to scan beat-by-beat time series (10,000 beats) of SABP and PI, searching for sequences of at least 4 consecutive beats in which increases in SABP were followed by PI lengthening (UP sequence) and decreases in SABP were followed by PI shortening (DOWN sequence) with a linear correlation higher than 0.8. The slope of the linear regression lines between SABP and PI was taken as a measure of spontaneous BRS.

*Behavioral analysis*

In order to have grooming behavior assessed, rats were observed during one hour following CeA bilateral microinjections of OT (0.5 μg or 1 μg) or SAL. Every 15 seconds, a point was scored if the animal displayed one of the following behaviors: body cleaning/washing, general grooming, scratching, licking of paws, grooming of head, grooming of face, grooming of anogenital region or tail, or grooming of another region of the body. The mean number of points in a 60 min interval is called the grooming score [5].

We also used the behavioral sequence flowchart method, a high-precision neuroethological approach, to analyze the behavioral responses in the SAL+OT and OTA+OT groups. With this method, we can check for the flux of statistical associations between pairs of behaviors (dyads). After digitalization, videos were analyzed and a behavior dictionary containing all observed behaviors was built. The construction of this dictionary was based on previous studies from our laboratory [6]. The flowcharts were divided into four time periods of 5 minutes each: the first comprises minutes 0 and 5, the third comprises minutes 45 and 50, and the fourth comprises minutes 55 and 60. The second period (correspondent to the behavioral peak) comprised different 5 minutes intervals for each treatment and animal to avoid selection bias. The criteria used are as follow: (1) possible intervals were chosen between minutes 20 and 35, as this was the period in which statistical difference was found in the grooming score (see Figure 2A) and the interval chosen was the one with the highest grooming score for each animal; (2) since the flowchart method analyses complete behaviors, if the animal presented a behavior in the exact minute that limits the grooming score analysis (for example, 25, 30, 35 and so on), the digital video was rewinded and the beginning of the 5-min interval was made coincident with that behavior.

*Experimental protocols*

Considering that in this first group, bilateral microinjections of OT (0.5 μg) into the CeA produced hypergrooming behavior but did not change cardiovascular and respiratory parameters, in the next step, we then tested the dose of 1 μg of OT [7], which was effective in producing simultaneous behavioral and cardiorespiratory responses. The peptide solution was freshly prepared just before being injected and it was diluted in sterile saline (NaCl 0.9%). MABP and HR values were continuously recorded for 10 min before and 60 min after bilateral microinjections of OT (0.5 μg or 1 μg) or SAL into the CeA. MABP or HR changes (respectively ∆MABP and ∆HR) sampled at 5 min intervals. Points sampled during the 10 min before microinjections of OT or SAL were used as control baseline value.

The involvement of oxytocin receptors on the behavioral, respiratory and cardiovascular responses to bilateral microinjections of OT into the CeA was evaluated in two distinct groups of rats: a) bilateral microinjections of OT (1 μg) into the CeA 10 min after bilateral microinjections of a selective oxytocin receptor antagonist (OTA) or bilateral microinjections of OT (1 μg) into the CeA 10 min after bilateral microinjections of vehicle (saline 0.9%). The dose of OTA (1 µg /200 nL) microinjected into the CeA was based on a previous study from our laboratory [6].

The involvement of oxytocin receptors in the CeA on the maintenance of MABP and HR was evaluated by the quantiﬁcation of these parameters before (control) and 9 min after bilateral microinjections of OTA or saline into the CeA (immediately before OT microinjections). All experimental protocols were performed between 12:00 and 18:00 hours.

*Histological analysis*

At the end of each experiment, animals were then deeply anesthetized with sodium thiopental (Abbott, North Chicago, IL, USA) 60 mg/kg and transcardially perfused with the aid of an infusion pump with 100 mL of saline phosphate buffer 0.01M (PBS, pH 7.4), followed by 300 mL of 4% paraformaldehyde (Acros Organics, Geel, Belgium). Brains were removed and post-fixed for about 2 h in paraformaldehyde 4 %, then cryoprotected in 20 % sucrose for 1–2 days at 4^º^C before being frozen in isopentane and sliced into 40-μm-thick coronal sections on a cryostat (Micron-Zeiss HM-505-E; Walldorf, Germany). Brain sections were mounted on gelatin-subbed glass slides and stored at -20ºC until further processing. Nissl staining was used to reveal correct cannula placement and the microinjections. For data analysis only the rats with the centers of the microinjections sites located bilaterally in the CeA were considered in the group with positive histology (group inside CeA). The rats brains in which the centers of the microinjections sites were located outside CeA (optic tract, basolateralamygdaloid nucleus and lateral globuspallidus) were considered in the group with misplaced microinjections (group outside CeA).

**Supporting Results**

*Histological analysis*

Figure S1A is a photomicrograph of a coronal section of the brain of one rat, representative of the group, showing the sites of bilateral microinjections of OT 0.5 μg, OT 1μg or SAL. Figures S1B-S1C indicate the sites of the centers of bilateral microinjections of OT 0.5 μg, OT 1μg or SAL into the CeA of rats with positive histology and the sites of microinjections outside of CeA. In experimental groups, only the animals that received bilateral microinjections of OT were considered in the data analysis, once those unilateral microinjections of OT 0.5 or 1 μg inside or outside CeA produced no significant changes on the behavioral, cardiovascular and respiratory parameters of animals.

Figure S4 illustrates behavioral sequences of the SAL+OT and OTA+OT groups using the detailed neuroethological approach. Four observational windows are represented: 0 to 5 min., behavioral peak, 45 to 50 min. and 55 to 60 min. Note in panel 0 to 5 minutes of the SAL+OT group (Figure S4B) the presence of orofacial automatisms and wet dog shakings (yellow symbols, WDS) linked to grooming of head (GRH; red symbol), as well as the clear cluster of GRH, licking of claws (LIC) and grooming of face (GRF; red symbols; Figure S4B). This effect was not observed in the same observational window of the OTA+OT group (Figure S4C). Both groups displayed significant interactions between orofacial automatisms (yellow symbols), exploratory activity clusters (blue symbols) and grooming behaviors (red symbols) during the behavioral peak (Figures S4B-S4C). However, when we verify the interactions between behaviors in the behavioral peak of the OTA+OT group there is a substantial reduction of frequency and duration in the exploratory activity and grooming behavior clusters. In addition, we observed in the SAL+OT group a small cluster of orafacial automatisms (yellow symbols; Yawn (YA) and masticatory movements (MT) linked to grooming behaviors (red symbols; grooming of genitalia (GRG) and GRH). At the same time in the OTA+OT group only one orofacial automatisms behavior (WDS) was linked to exploratory activity (SC; Figures S4B-S4C). We also confirmed that pretreatment with OTA resulted in an incomplete blockade of OT-induced hypergrooming. When we compared the 45 to 50 min. panels in the SAL+OT group, we observed a cluster of behaviors involving LIC linked to GRF and left scratch (SCRL) linked to licking of the posterior left claws (LCL2; Figure S4B). In the OTA+OT group, we observed an interaction only between right scratch (SCRR) and licking of posterior right claws (LCR2; Figure S4C), but none between the orofacial automatisms, exploratory activity and grooming behaviors. In the fourth period (55-60 min), only the SAL+OT group exhibited LCL2 linked to the exploratory activity MT, showing that this group exhibits grooming behavior even at 60 min after the microinjections.

**Supporting References**

1. Paxinos G, Watson C (2005) The rat brain in stereotaxic coordinates. New York, NY, USA: Elsevier Academic Press. 228 p.

2. Dias da Silva Valdo J, Viana Públio CC, de Melo Alves R, Fazan R Jr, Ruscone TG, et al. (2002) Intravenous amiodarone modifies autonomic balance and increases baroreflex sensitivity in conscious rats. Auton Neurosci 95: 88–96.

3. Fazan R Jr, Huber DA, Silva CAA, Dias da Silva VJ, Salgado MCO, et al. (2008) Sildenafil acts on the central nervous system increasing sympathetic activity. J Appl Physiol 104: 1683–1689. doi:10.1152/japplphysiol.01142.2007.

4. Di Rienzo M, Bertinieri G, Mancia G, Pedotti A (1985) A new method for evaluating the baroreflex role by a joint pattern analysis of pulse interval and systolic blood pressure series. Medical and Biological Engineering and Computing 23: 313–314.

5. Gispen WH, Wiegant VM, Greven HM, de Wied D (1975) The induction of excessive grooming in the rat by intraventricular application of peptides derived from ACTH: structure-activity studies. Life Sci 17: 645–652.

6. Marroni SS, Nakano FN, Gati CDC, Oliveira JAC, Antunes-Rodrigues J, et al. (2007) Neuroanatomical and cellular substrates of hypergrooming induced by microinjection of oxytocin in central nucleus of amygdala, an experimental model of compulsive behavior. Mol Psychiatry 12: 1103–1117. doi:10.1038/sj.mp.4002015.

7. Drago F, Bohus B (1981) Hyperprolactinemia-induced excessive grooming in the rat: time-course and element analysis. Behav Neural Biol 33: 117–122.
